# Temporal and diurnal variation in social media posts to a suicide support forum

**DOI:** 10.1186/s12888-021-03268-1

**Published:** 2021-05-19

**Authors:** Rina Dutta, George Gkotsis, Sumithra Velupillai, Ioannis Bakolis, Robert Stewart

**Affiliations:** 1 Department of Psychological Medicine, School of Academic Psychiatry, King’s College London, IoPPN, PO Box 84, 3rd Floor East Wing, Room E3.07, De Crespigny Park, London, SE5 8AF UK; 2 South London and Maudsley NHS Foundation Trust, London, UK; 3 School of Electrical Engineering and Computer Science, KTH, Stockholm, Sweden

**Keywords:** Suicide, Suicide timing, Social media, Diurnal rhythms, Temporal pattern

## Abstract

**Background:**

Rates of suicide attempts and deaths are highest on Mondays and these occur more frequently in the morning or early afternoon, suggesting weekly temporal and diurnal variation in suicidal behaviour. It is unknown whether there are similar time trends on social media, of posts relevant to suicide. We aimed to determine temporal and diurnal variation in posting patterns on the Reddit forum SuicideWatch, an online community for individuals who might be at risk of, or who know someone at risk of suicide.

**Methods:**

We used time series analysis to compare date and time stamps of 90,518 SuicideWatch posts from 1st December 2008 to 31st August 2015 to (i) 6,616,431 posts on the most commonly subscribed general subreddit, AskReddit and (ii) 66,934 of these AskReddit posts, which were posted by the SuicideWatch authors.

**Results:**

Mondays showed the highest proportion of posts on SuicideWatch. Clear diurnal variation was observed, with a peak in the early morning (2:00–5:00 h), and a subsequent decrease to a trough in late morning/early afternoon (11:00–14:00 h). Conversely, the highest volume of posts in the control data was between 20:00–23:00 h.

**Conclusions:**

Posts on SuicideWatch occurred most frequently on Mondays: the day most associated with suicide risk. The early morning peak in SuicideWatch posts precedes the time of day during which suicide attempts and deaths most commonly occur. Further research of these weekly and diurnal rhythms should help target populations with support and suicide prevention interventions when needed most.

**Supplementary Information:**

The online version contains supplementary material available at 10.1186/s12888-021-03268-1.

## Background

Studying public mental health through social media is a burgeoning area of research [1, 2] which is changing the way in which we understand mental health indicators, such as social isolation and suicidality, in the general population [3, 4]. Direct contact on social media has enabled sharing of concerns beyond the boundaries of face-to-face interactions and connecting people who would otherwise not have communicated [5]. In 2018, a study of more than 4000 UK and US adults found that over 80% were using one or more social networking sites [6], making it possible to study population-level disclosure of mental health symptoms.

Reddit is a popular public online forum covering a diverse range of topics, featuring a user-voting system to rank posts, comments, and links within its sub-communities (known as subreddits). Most Reddit users (54%) come from the USA, with the UK ranking second and Canada third [7]. SuicideWatch is a subreddit where people post about their suicidal thoughts (or about suicide-related issues regarding someone they know) to receive feedback and support from the community and it should be emphasised that posts do not necessarily reflect authorship by people experiencing suicidal ideation. Nevertheless, because this is a highly sensitive topic, human moderators make sure that comments left for a post are not abusive [8]. Work to date includes attempts to automatically identify helpful comments [8] and changes in posting activity after high-profile suicides [9].

Weekly variation in suicide mortality has been investigated in many studies in both the US and UK, with the majority reporting peaks on Mondays declining to weekend lows [10–12], although others have noted a peak in the middle of the week with higher suicide rates on Wednesdays [13]. Diurnal patterns appear to vary by gender, age, and chronological time period, with one Italian study showing peaks in both suicide attempts and deaths in the morning and early afternoon [14]. However findings may differ by country and subpopulation, as indicated by a recent study national data from 1974 to 2014 in Japan [15]. This showed suicide by middle-aged males was most frequent in the early morning especially on Mondays after the end of Japan’s high growth period. Large midnight peaks in suicide deaths were also observed among young and middle-aged males. The proportion of early morning suicide deaths by young and middle-aged males increased as the country’s unemployment rose. Females and elderly males were more likely to die by suicide during the day than at night. Interestingly the authors note the limitation that they studied the time of death, and of course this will be later than the attempt, and indeed the antecedent suicidal crisis when social media posting might occur.

We investigated whether there are similar weekly and daily trends in the way that Reddit is used by those posting to SuicideWatch with their public user accounts, and with anonymous accounts: so-called ‘throwaway accounts’, (which we shall term ‘anonymous SW’). These accounts may reveal more sensitive information and socially unacceptable feelings owing to their anonymity [16]. We compared how these posting behaviours are different from general Reddit use by all users, and by those authors who post with identifiers on SuicideWatch.

## Methods

### Sample

We acquired a complete, publicly available, Reddit data dump for the period between December 2008 and August 2015 (https://redd.it/3mg812). The acquired and indexed dataset contains 196 million posts across thousands of different fora (subreddits). Using a set of carefully curated keywords related to mental health [17], we had previously identified subreddits associated with mental health [18, 19]. The subreddit data used in this study is from SuicideWatch (90,518 posts from 42,723 authors), a forum dedicated to the online support of users with suicidal ideation, or who know someone at risk. All posts to SuicideWatch in the specified timeframe were analysed and no exclusion criteria were applied. We refer to this dataset as ‘SW’ hereafter. Overall, Reddit users comprise younger males, with poll data showing 64% of Reddit users are between 18 and 29 years of age and 69% of American Reddit users are male [7]. However Reddit users only provide a username, password and email address to sign up, so we cannot evaluate the age and sex distribution of Reddit authors contributing posts to the datasets in our study, nor study changes in demographics over time.

A subset of this subreddit is comprised of authors that contain the keyword ‘throw’, who can be considered to have ‘anonymous SW accounts’ (5882 authors, 13.8% of the SW authors). The use of the keyword ‘throw’ has been shown to be a high precision technique for identifying anonymous accounts [16, 20]. Anonymous accounts are multiple temporary identities permitted by Reddit. They are perceived to retain anonymity by many users and allow authors to hide their overall activity in other subreddits [16]. It was postulated that these posts might exhibit different patterns compared to those with author-identifiers. This data subset is referred to as ‘Anonymous SW’ hereafter.

To identify potential control datasets, we extracted the author-identifiers in SW and looked up their posts elsewhere, excluding their posts to other mental health-related subreddits [18]. We selected AskReddit, a subreddit which contains posts about a multitude of topics and with the highest number of subscribers (over 15 million, and 1.4 million authors in our data): http://redditmetrics.com/r/AskReddit. It was also the most popular subreddit among the SW authors (66,934 posts and 8065 authors).

We identified two control datasets: (i) AskReddit posts from all AskReddit authors (‘AR’) and (ii) the subset of AskReddit which were posted by SW authors, referred to as AskReddit-SW author posts (and the dataset as ‘AR-SW’) hereafter. (Refer to GitHub repository - https://github.com/KCL-Health-NLP/temporal_variation_sw - for a more detailed description of the data analysis process).

Table 1 summarizes the number of post authors and numbers of posts in the four datasets: Anonymous SW is a subgroup of SW and AR-SW is a subgroup of AR. The “posts per author” column was calculated for account users with unique IDs. There were only *n* = 108 deleted posts in the dataset and we are unable to deduce the number of unique deleted authors.

**Table 1:** Descriptive Statistics of the four datasets used in this study

Source subreddit	Total no. of post authors	Total no. of posts	Posts per author Mean (s.d.); median
SuicideWatch (SW)	42,723	90,518	1.25 (1.32); 1
Anonymous SW	5882	6557	1.11 (0.71); 1
AskReddit (AR)	1,438,227	6,616,431	2.98 (8.48); 1
AskReddit-SW authors (AR-SW)	8065	66,934	8.30 (30.00); 3

### Datasets

To determine the timing of post publication, we leveraged a specific field found within each post that records the local time of the author, as documented by the JSON API of Reddit (see field “created” at https://github.com/reddit/reddit/wiki/JSON). Furthermore, in order to aggregate and extract the temporal characteristics of the posting behaviour, we considered a time window of 1 week. Starting from Monday through Sunday, we extracted the timestamp of posts and kept both hour of the day and day of the week according to the author’s local time. In order to address weeks where limited activity occurred (and might therefore introduce measurement error as the signal might become skewed), we only kept weeks during which more than 200 posts had been published. This led to a reduction of between 0.0001% (AskReddit) and 0.55% (Anonymous SW) of the overall posts made. The final number of weekly observations varied for each data source, being 341 weeks for SW, 246 for Anonymous SW, 391 weeks for AskReddit and 354 weeks for AskReddit (SW-authors).

For each weekly observation, we counted the number of posts made in the corresponding time frames (e.g. for the “hour of the day” analysis, we counted posts made during each hour, between minute 00 to minute 59 in all 7 days of the week, and for the “day of the week” analysis we counted posts made on each different day). As the total number of observations differed in each dataset over the entire period (generally increasing in number over time), it was necessary to produce normalised figures. We produced the percentages for each week using the total number of posts made by the corresponding set of authors in the observed week. For instance, a percentage value of 5% for AskReddit for a particular hour of the day refers to the percentage of the total number of posts on AskReddit during that hour of the day throughout the whole of the “observed week”.

### Statistical analyses

We explored temporal associations of days of the week and hours of the day between SW, AskReddit, AskReddit (SW-authors) and Anonymous SW with the use of fractional response regression models, which are appropriate to use when the outcome of interest is a proportion which is bounded between 0 and 1 (e.g. proportion of posts in SW) [21]. We chose fractional logistic regression models, as they can tackle inference issues which is a possibility with values close to the boundaries. Generalised additive models (GAMs) [22] were also employed to test any non-linear associations of days of the week and hours of the day between SW and the three other datasets. GAMs are flexible extensions of Generalized Linear Models (GLMs). In GAM the link function includes flexible functions of smoothing splines, where the amount of smoothing applied to each predictor is controlled by the user according to a quantity known as the equivalent degrees of freedom (df). We used a Poisson additive model for these data and allow each function to have 3 degrees of freedom (df) but also chose values 1, 3 and 10.

We also estimated the mean difference in proportions (MD) and corresponding 95% confidence intervals (95% CI) of SW compared to AskReddit and AskReddit (SW-authors) for six-hour intervals within a 24-h day - chosen a priori as early hours of the morning (midnight to 05:59 h), morning (06:00–11:59 h), afternoon (noon-17:59 h) and night (18:00–23.59 h) - for the complete time period under study.

## Results

SW authors had temporary identities (anonymous accounts) in 5882/42,723 (13.8%) cases.

It is immediately apparent in Table 1 that AR-SW authors posted disproportionally higher numbers of posts (median *n* = 3; compared to *n* = 1 posts for authors in all other datasets). However, the outcome of Mann-Whitney U tests between each pair of datasets was *p* < 0.001, indicating strong disparity between each pairwise comparison, with anonymous SW authors posting the least per author, followed by SW authors, then AR and highest levels of posting by AR-SW authors.

### Day of the week results

**Fig. 1:**
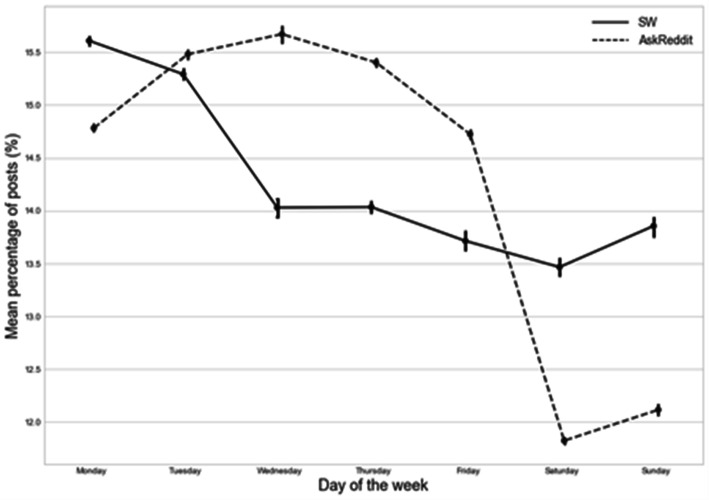
Trajectory of mean percentage of posts (with 95% CI) by group (SuicideWatch (SW) and AskReddit authors) according to day of the week

Figure 1 illustrates the peak in SW posts on Mondays (solid line): mean 15.61% (95%CI 15.57–15.65); compared to mean 14.78% (95%CI 14.75–14.82) posts for AskReddit authors (dashed line). Whereas the peak in AskReddit posts occurred on Wednesday: mean 15.67% (95%CI 15.59–15.75). The mean percentage of posts was lowest on Saturdays for both source subreddits: mean 13.47% (95%CI 13.38–13.56) for SW compared to mean 11.82% (95%CI 11.79–11.85) for AskReddit authors. For clarity only the trajectory of mean percentages of posts for SW and AskReddit are summarised in the figure (Anonymous SW followed a similar trajectory to SW and AskReddit (SW-authors) followed the AskReddit trend. These have been omitted from Fig. 1 to allow the overall trends to be more clearly observed).

Users of SW had the highest probability of posting to this subreddit on Mondays compared to all other days of the week (ratios ranged from 1.025 (95%CI: 1.020–1.030) to 1.189 (95% CI: 1.180–1.198); all *p* ≤ 0.001 - see Online Table 1). They also showed higher probability of posting on Tuesdays compared all other days of the week, apart from Monday (ratios ranged from 1.106 (95%CI: 1.097–1.115) to 1.160 (95% CI: 1.151–1.170); all *p* ≤ 0.001 - see Online Table 1). There was no evidence for a difference between posts on SW on Wednesdays compared to Thursday as the baseline. However Wednesdays compared to Friday, Saturday and Sunday showed elevated ratios. Thursdays compared to Friday, Saturday and Sunday had similar estimates. Fridays compared to Saturday has a slightly elevated ratio of probability 1.021 (95% CI: 1.011–1.032) p ≤ 0.001, and compared to Sundays had a lower ratio of probability 0.988 (95% CI: 0.978–0.999) *p* = 0.029. Saturday also showed a lower ratio of probability compared to Sunday 0.968 (95% CI: 0.958–0.977) *p* ≤ 0.001. (Refer to Online Table 1 for all ratios of probability and 95% CIs by day of the week).

### Time of the day results

**Fig. 2:**
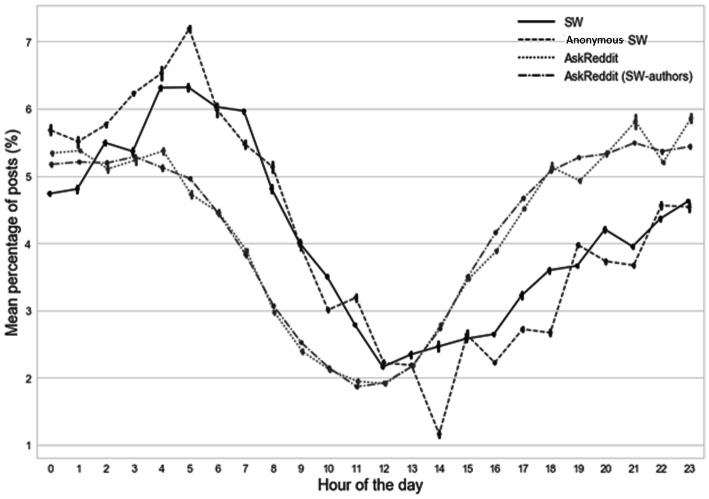
Trajectory of mean percentage of posts (with 95% CI) by group (Suicide Watch (SW), Anonymous SW and control groups: AskReddit (all users) and AskReddit (SW-authors)) according to hour of the day, across all days of the week

We explored if there was a non-linear association between the timing of Reddit posts by hours of the day, when comparing each of the four different datasets with each other, using generalised additive (GAM) models. Within each dataset, non-linear associations were not observed between suicide related posts and hours of the day (*p*-values > 0.05) (this is something expected from observation of the descriptive graphs, as the four dataset trajectories are following the same hourly pattern in Fig. 2).

Figure 2 shows a clear diurnal variation in the distribution of SW posts, with an upward trend visible in the early hours of the morning (2:00–5:00 h; highest mean percentage at 5:00 h; 6.32, 95%CI (6.26–6.37%)), and a subsequent decrease to a trough in late morning / early afternoon (11:00–14:00 h; lowest mean percentage at 12noon; 2.16, 95%CI (2.15–2.17%)), whereas the same authors made the highest volume of posts on AskReddit between 20:00–23:00 h (highest mean percentage at 23:00 h; 5.86, 95%CI (5.79–5.93%)), which is also the time period when all AskReddit authors are more likely to post (highest mean percentage at 21:00 h; 5.49, 95%CI (5.48–5.51%)).

The mean percentage of posts in SW was higher between midnight and noon and lower between noon and midnight expressed as a ratio of both AskReddit control groups (as shown in Online Figure 1). The six-hour interval when the ratio of SW posts far exceeded the AskReddit posting level was between 6 am-12 pm.

## Discussion

We found strong evidence for variation by day of the week and time of day for posts by Reddit-users to the subreddit SW, both absolute and when compared to the same authors’ activity on the most popular ‘non-mental health’ subreddit AskReddit as well as all Reddit-users’ activity on AskReddit.

Overall, higher levels of posting in AskReddit by SW authors could indicate that those posting to SW are higher intensity users of the Reddit platform, posting to other subreddits too, and finding it helpful to seek support via the SW forum when in crisis.

Monday was associated with the highest SW posting levels compared to other days of the week. The trend over weekly cycles closely follows weekly suicide risk patterns reported by most previous studies, with a peak on Monday, a decreasing risk over the course of the week [23], and the lowest incidence at the weekends [24]. Therefore the weekly cycle analysis provides face validity that relevant social media postings may track the days most associated with suicide risk.

The diurnal variation in SW posting that we present is as markedly compelling as a biological phenomenon such as diurnal cycling of cortisol levels – a hormone well-known for its effect on mood [25]. Most strikingly the volume of SW Reddit posts peak immediately prior to hours of the day when suicide attempts and suicide incidence are at their highest in many countries. This raises the possibility that posting at such times could be averting a crisis for some individuals, or that this could be a time when interventions might be made more accessible.

Given that the timing of most SW posts is in the early hours of the morning (peak 05:00 h), contrasting with the highest volume of AskReddit posts being in the late evening (peak 21:00 h), sleep disturbance may represent one potential modifiable risk factor both for suicidal thoughts being expressed through social media and suicidal behaviours [26, 27]. From a clinical standpoint, patients experiencing insomnia in the early hours are much more able to identify when they have an issue with their sleep [28] and can be more willing to reveal this to their doctors than a potential mental health concern [29]. Given that greater social media use has been shown to be significantly associated with disturbed sleep [30, 31] as well as anxiety, depression and low self-esteem [32, 33] this may be a useful emerging area when recording the history of patients with a mental health issue. Therefore reducing specific night-time social media use might be a focus of targeted psychological interventions for some individuals.

It would seem likely therefore that there are several potential competing hypotheses as to the meaning of the results. For example: in the early hours of the morning (1) suicidal individuals are communicating their suicidal plan or intent, (2) are using social media as a means of distraction, social engagement, or help-seeking to avert a potential crisis, (3) are posting because those at risk of suicide often have mental health issues characterised by circadian rhythm dysfunction, leading to poor nocturnal sleep, (4) engage with the Reddit platform for some other reason, or (5) a combinations of these or other factors. Recommendations for how and whether to intervene will differ depending on which of these is occurring and it will likely vary by different groups within the population.

In their work, Pavalanathan and De Choudhury (2015) report a 9% presence of anonymous authors, whereas in our dataset we observed a presence of almost 14% (5882/42,723) suggesting more temporary identities are created to discuss thoughts or emotions around suicide. The authors may be revealing more sensitive or personal information and socially unacceptable feelings reflected in their anonymity.

### Strengths and limitations

The key strengths of this study are the clear weekly and diurnal rhythms revealed simply using the timing of postings of Reddit data. No pre-processing steps were required, nor did the semantic content of the posts have to be analysed for these to be recognised as strong signals, similar to those observed for socialising propensity using online gaming data, which showed the highest probability of making social connections at night (between 20:00 h and midnight) [34].

That said, we do not gain knowledge about factors underlying people’s choices in posting to the subreddit SW. Only the timing of the posts was analysed, not the content, and not all posts were necessarily ‘suicide-related’. If someone posted something not suicide-risk related (e.g. information about a support service), this would have been included in the same way as a post reaching out for support in a suicidal crisis. The data used in our study were also based on the time of posting and therefore assume that the author is writing contemporaneously. Content analyses might reveal that different lexicon and linguistic attributes were used at different times and this could be a useful extension to the study. It was not possible to investigate author characteristics (e.g. age or sex) owing to the anonymity of the data and therefore it is not possible to ascertain the representativeness of the sample with regard to all Reddit users.

A recent study of the Reddit data dump [35] has found substantial missing observations, which could affect research validity in this and all other publicly crawled social media datasets. However this is more likely to be an issue when user histories are studied or network analysis is conducted, rather than simply studying the counts and timing of posts. Furthermore we could not investigate associations between posts and mental health outcomes or suicide outcomes, so the results are observing a pattern of association without directly linking it with the outcome.

The analysis we present is an aggregate over a long period of time across a large set of users, which reveals a clear overall pattern for the cohort of users. Individual patterns may of course differ enormously, particularly as many users publish just a few posts in this subreddit (which is intended for peer support during a crisis). We only studied original posts, not ‘comment’ postings from users, so multiple supportive comment posts in the same visit would not impact the aggregate results. Any analysis on an individual level would require a range of other considerations, including ethical ones, which were outside the scope of this study.

## Conclusions

Given comments on posts from users expressing suicidal thoughts can be written from any part of the world at any time, moderating SW and other sensitive fora in a timely manner can be challenging. However at a national level in countries with one time zone, (e.g. the UK), or at a State or regional level for countries with multiple time zones, (e.g. the USA or Russia), it is entirely plausible to envisage providing higher levels of moderation on social media at times of increased posting about suicidality and potentially developing links to online interventions or sources of support [36]. There is likely to be substantial variability between countries in terms of sources of moderators (e.g. from the voluntary/charitable sector, from support agencies) and it would be overly speculative and beyond the scope of this paper to make specific recommendations about the agency who should provide moderation/support, although research into what constitutes a helpful comment from a suicide prevention perspective is ongoing [8]. Also owing to the need for assured confidentiality on such internet fora, the potential association between SW posts and suicide attempts is unlikely to be conclusively investigated and any intervention would have to be justifiable as good practice.

In a societal context, the clear weekly and diurnal rhythms of propensity of posting to SW should enable improved access to online support or targeted messages during the temporal window in which there is an increased number of SW posts as identified in this study. Developed carefully these have the capacity to target otherwise unreachable populations and broker suicide prevention messaging and interventions when needed most.

## Supplementary Information

**Additional file 1: Online Table 1.** Subreddit posts in SuicideWatch according to different days of the week. Ratio (R) of probability of posting and 95% Confidence Intervals (95% CI) on each day of the week compared to each possible baseline day. **Online Figure 1.** Mean Difference (MD) and 95% Confidence Intervals (95% CI) in proportions of Suicide Watch (SW) compared to the 2 control groups: AskReddit (all users) (AR) and AskReddit (SW-authors) (AR-c). Horizontal axis represents the hours of the day in six-hour intervals, across all days of the week.

## Data Availability

Technical appendix and code available at https://github.com/KCL-Health-NLP/temporal_variation_sw and datasets available by request from the corresponding author.
