# Supplementary material for: Temporal and diurnal variation in social media posts to a suicide support forum
Source: BMC Psychiatry. 2021 May 19;21:259. doi: 10.1186/s12888-021-03268-1 (PMC8136175; doi:10.1186/s12888-021-03268-1)
Supplement: Supplementary file 1 — Additional file 1: Online Table 1. Subreddit posts in SuicideWatch according to different days of the week. Ratio (R) of probability of posting and 95% Confidence Intervals (95% CI) on each day of the week compared to each possible baseline day. Online Figure 1. Mean Difference (MD) and 95% Confidence Intervals (95% CI) in proportions of Suicide Watch (SW) compared to the 2 control groups: AskReddit (all users) (AR) and AskReddit (SW-authors) (AR-c). Horizontal axis represents the hours of the day in six-hour intervals, across all days of the week. [file 12888_2021_3268_MOESM1_ESM.docx]

**SUPPLEMENTARY FILE**

**Temporal and diurnal variation in social media posts to a suicide support forum**

Rina Dutta* FRCPsych, PhD 1,2, George Gkotsis PhD 1, Sumithra Velupillai PhD 1,3 , Ioannis Bakolis PhD 1, Robert Stewart FRCPsych, PhD 1,2

1. King’s College London, IoPPN, London, SE5 8AF, UK

2. South London and Maudsley NHS Foundation Trust, London, UK

3. School of Electrical Engineering and Computer Science, KTH, Stockholm

∗ Corresponding author

Dr Rina Dutta

Senior Clinical Lecturer / Consultant Psychiatrist

Department of Psychological Medicine, Division of Academic Psychiatry

PO Box 84 | Room E3.07 | 3rd Floor East Wing | IoPPN

King's College London | De Crespigny Park | London SE5 8AF

[rina.dutta@kcl.ac.uk](mailto:rina.dutta@kcl.ac.uk) | Tel +44 (0)7904 207378

Other author emails: [gkotsis@gmail.com](mailto:gkotsis@gmail.com); [sumithra.velupillai@kcl.ac.uk](mailto:sumithra.velupillai@kcl.ac.uk); [ioannis.bakolis@kcl.ac.uk](mailto:ioannis.bakolis@kcl.ac.uk); robert.stewart@kcl.ac.uk

Online Table 1 - Subreddit posts in SuicideWatch according to different days of the week.

Ratio (R) of probability of posting and 95% Confidence Intervals (95% CI) on each day of the week compared to each possible baseline day.

| Baseline  day | Mon  R  (95% CI) | Tues  R  (95% CI) | Wed  R  (95% CI) | Thurs  R  (95% CI) | Fri  R  (95% CI) | Sat  R  (95% CI) |
| --- | --- | --- | --- | --- | --- | --- |
| Tues | **1.025**  **(1.020-1.030)** |  |  |  |  |  |
| Wed | **1.133**  **(1.125-1.142)** | **1.106**  **(1.097-1.115)** |  |  |  |  |
| Thur | **1.133**  **(1.127-1.139)** | **1.106**  **(1.099-1.112)** | 1.000  (0.991-1.008)  p=0.924 |  |  |  |
| Fri | **1.164**  **(1.154-1.174)** | **1.136**  **(1.126-1.146)** | **1.027**  **(1.016-1.038)** | **1.027**  **(1.018-1.036)** |  |  |
| Sat | **1.189**  **(1.180-1.198)** | **1.160**  **(1.151-1.170)** | **1.049**  **(1.038-1.059)** | **1.049**  **(1.040-1.058)** | **1.021**  **(1.011-1.032)** |  |
| Sun | **1.150**  **(1.141-1.159)** | **1.122**  **(1.113-1.132)** | **1.015**  **(1.004-1.025)**  **p=0.005** | **1.015**  **(1.006-1.024)** | **0.988**  **(0.978-0.999)**  **p=0.029** | **0.968**  **(0.958-0.977)** |

**All p-values≤0.001** except where stated

Online Figure 1: Mean Difference (MD) and 95% Confidence Intervals (95% CI) in proportions of Suicide Watch (SW) compared to the 2 control groups: AskReddit (all users) (AR) and AskReddit (SW-authors) (AR-c). Horizontal axis represents the hours of the day in six-hour intervals, across all days of the week


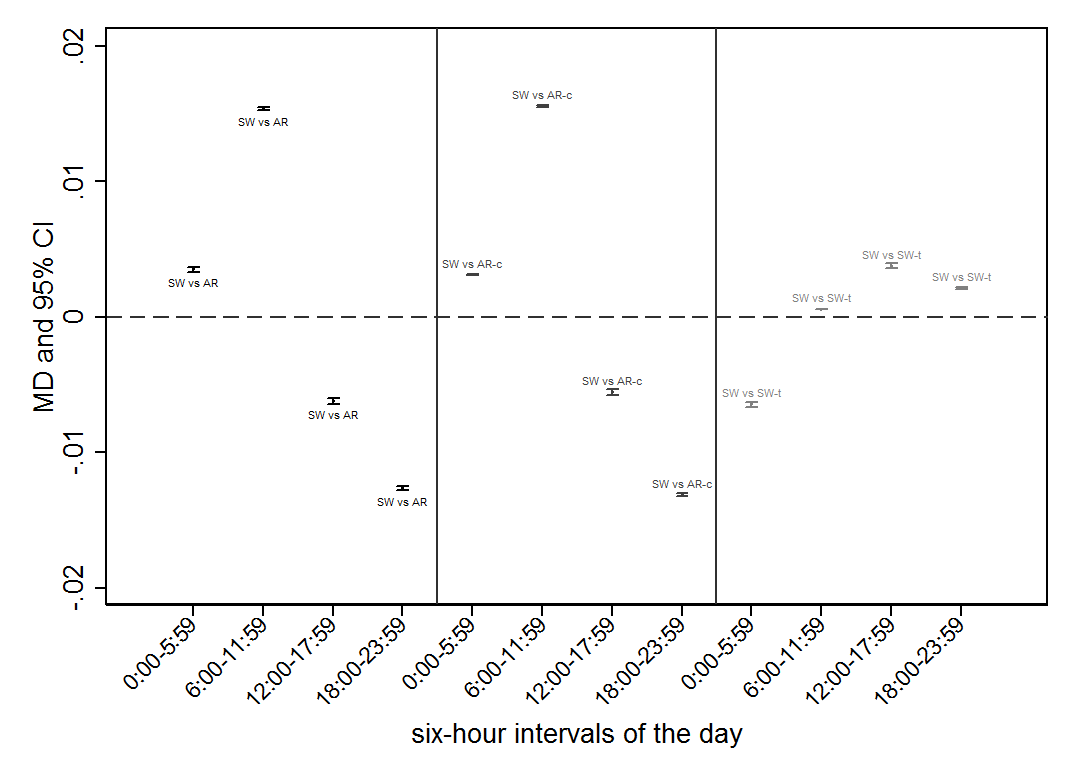


six-hour interval of the day
